# Draft Genome Sequence of Subantarctic *Rhodococcus* sp. Strain 1139

**DOI:** 10.1128/genomeA.00090-17

**Published:** 2017-04-06

**Authors:** Akhikun Nahar, Anthony L. Baker, Michael A. Charleston, Margaret L. Britz

**Affiliations:** a Tasmanian Institute of Agriculture, University of Tasmania, Hobart, Australia; b School of Physical Sciences, University of Tasmania, Hobart, Australia; c Faculty of Veterinary and Agricultural Sciences, The University of Melbourne, Melbourne, Australia

## Abstract

The draft genome sequence of subantarctic *Rhodococcus* sp. strain 1139 is reported here. The genome size is 7.04 Mb with high G+C content (62.3%) and it contains a large number of genes involved in lipid synthesis. This lipid synthesis system is characteristic of oleaginous *Actinobacteria*, which are of interest for biofuel production.

## GENOME ANNOUNCEMENT

Nonpathogenic *Rhodococcus* species have long been recognized as attractive targets for industrial purposes because of their diverse metabolic capacity (1–3). They occupy a variety of ecological niches, temperature zones, and survive long periods in soil (1). The genus *Rhodococcus* is a member of the supergeneric taxon mycolata under the phylum *Actinobacteria*, members of which uniquely synthesize mycolic acids in the lipid-rich Gram-positive cell wall. This taxon includes the genera *Rhodococcus*, *Corynebacterium*, *Mycobacterium*, *Dietzia*, *Gordonia*, *Williamsia*, *Segniliparus*, *Skermania*, *Tsukamurella*, and *Nocardia* (4–6). Many species accumulate lipids, including triacylglycerols, as storage compounds (7) which are important for biofuel development from inexpensive carbon sources (8, 9).

*Rhodococcus* sp. 1139 was originally isolated in 2011 from soils from Macquarie Island, a subantarctic region of Australia, and stored in the University of Tasmania Antarctic Culture Collection. This strain is an aerobic, psychrotrophic, short Gram-positive rod. 16SrDNA sequencing (DNA extracted using the Bioline Isolate II genomic DNA kit, Australia) and BLAST analysis showed 100% similarity with *Rhodococcus* sp. YL-1, *Rhodococcus* sp. 008, *R. erythropolis*, *R. baikonurensis*, and *R. qinshengii*.

High molecular weight genomic DNA was obtained using a modified extraction method originally described by V. Lévy-Frébault et al. (10). Miseq Illumina (Macrogen, Korea) technology was used to sequence the genome. A total of 5,733,610 reads and 1,713,692,749 total bases were obtained. ABySS software (11) was used to assemble the raw data and the assembled sequence contained 187 contigs with length ≥200 bp.

The assembled data was annotated using RAST (12) and the NCBI Prokaryotic Genome Annotation Pipeline (https://www.ncbi.nlm.nih.gov/genome/annotation_prok/). This analysis identified 6,622 coding sequences (CDSs), 6,405 genes, and 217 pseudogenes. The genome has three complete rRNAs, 52 tRNAs, and three noncoding RNAs (ncRNAs). The genome content is generally consistent with *R. erythropolis* strain JCM 6824 (NCBI reference sequence: NZ_DF836179.1) and *R. qingshengii* JCM 15477 (NCBI reference sequence: NZ_LRRJ00000000.1), where whole-genome shotgun sequencing showed 7.023 Mb with 6,555 genes and 6,341 CDSs and 7.26 Mb with 6,573 genes and 6,573 CDSs, respectively. The closet neighbors found from RAST SEED Viewer 2 were *R. erythropolis* PR4 (score 530), *R. erythropolis* Sk121 (score 518), *R. jostii* RHA1 (score 504), and *R. opacus* B4 (score 489). Genetic similarities were compared using the Average Nucleotide Identity (ANI) tool (http://enve-omics.ce.gatech.edu/ani/) (13): >98% identity was shown with *Rhodococcus* sp. YL-1, *Rhodococcus* sp. 008, and *R. gingshengii* CS98.

SEED Viewer 2 identified 557 genes involved in carbohydrate metabolism, 25 in nitrogen metabolism, 350 in fatty acid, lipid, and isoprenoid biosynthesis and metabolism (including one 9.31 Kb FASI gene and multiple copies of FASII and polyketide synthase and regulatory genes, the latter likely involved with mycolic acid synthesis), 157 genes involved in stress responses, and 93 in metabolism of aromatic compounds. As this bacterial strain has a high number of genes involved in carbon metabolism and lipid biosynthesis, it may have potential for biofuel development and for studying mechanisms of adaptation to survival at low temperatures.

### Accession number(s).

This whole-genome shotgun project has been submitted to DDBJ/EMBL/GenBank under the accession number LWHK00000000.
